# Enhancing the growth and essential oil components of *Lavandula latifolia* using *Malva parviflora* extract and humic acid as biostimulants in a field experiment

**DOI:** 10.1038/s41598-024-82127-x

**Published:** 2025-01-04

**Authors:** Mervat El-Hefny, Mahmoud Khattab Hussien

**Affiliations:** Department of Floriculture, Ornamental Horticulture and Garden Design, Faculty of Agriculture (El-Shatby), Alexandria University, Alexandria, 21545 Egypt

**Keywords:** Biostimulant, Essential oil, Humic acid, Lavender, *Malva parviflora*, Plant extract, Chemical biology, Physiology, Plant sciences

## Abstract

Natural extracts as biostimulants have the potential to enhance the productivity and growth of many medicinal and aromatic plants. This study aimed to enhance the growth, and essential oil (EO) content, as well as composition of *Lavandula latifolia* Medik. by using *Malva parviflora* L. extract (ME) as a biostimulant in combination with humic acid (HA) in a field experiment in two successive seasons of 2022 and 2023. The phenolic, flavonoid and water-soluble vitamins of the ME were analyzed using an HPLC. The protein amino acids of the ME were identified by an amino acid analyzer. The prepared concentrations of HA (0, 1, 2, and 4 g/L) were applied to the soil. While, they for ME (0, 2, 4, and 6 g/L) were added as a foliar spray. The EO compositions collected from the leaves of the treated *L. latifolia* plants were subjected to the hydro-distillation method and analyzed using GC-MS. The most prevalent vitamins found in ME were vitamin B12, vitamin C, and folic acid. Besides, several phenolic compounds were found in ME, such as catechol, cinnamic acid and syringic acid, while flavonoid chemicals, such as luteolin and quercetin. Also, alanine, ammonia, aspartic acid, glutamic acid, glycine, and tyrosine were the ME’s most prominent nitrogenous and amino acid components. The most effective treatments of HA and ME on the plant height, the number of branches/plant, and plant fresh weight were 4 + 6 g/L and 4 + 2 g/L for leaf area and chlorophyll content, it was 4 + 4 g/L; and for EO percentage were 4 + 0 g/L, 2 + 0 g/L, and 4 + 4 g/L, compared to the control treatment for each characteristic. The main EO compounds eucalyptol, camphor, *α*-pinene, *β*-pinene, Δ-elemene, germacrene D-4-ol, isoborneol, *β*-caryophyllene oxide, and tau.-cadinol identified in the leaves were found in the range of 28.74–46.19%, 15.34–30.49%, 3.39–7.16%, 0–5.08%, 0–5.18%, 0–3.20%, 0–3.31% and 0–3.40%, respectively. It can be concluded that a combination treatment of HA and ME as natural biostimulant compounds at 4 + 4 g/L could be recommended for good plant growth, and EO quantity of *L. latifolia* plants.

## Introduction

Spike lavender (*Lavandula latifolia* Medik.) is an aromatic shrub belonging to the family Lamiaceae, native to the Mediterranean region and cultivated worldwide, which produces the spike lavender essential oil (EO) of commerce^1^. The most important Spanish EOs produced commercially from spike lavender^2^. Spike lavender and other lavender species have been traditionally used in perfumes and cosmetics^3,4^. In addition, medical uses have also been reported, including anti-inflammatory, anti-hypertensive, antiseptic, antispasmodic, healing, and sedative properties^5,6^. *L. latifolia* EO, with its main compounds including linalool, 1,8-cineole, and camphor, showed synergistic anti-microbial effects in combination with camphor^7^.

Nowadays, the pure EO of *L. latifolia* is used in aromatherapy^8^. The pleasant fragrance, physiological effects, and commercial significance are due to their EOs, whose highly variable composition depends on genetic, environmental, and processing factors^9^. Spike lavender EO is very popular and shows anti-bacterial, anti-fungal, anti-depressive, and highly effective properties for insect bites^10^.

The chemical composition of lavender oil varies based on the species. The variations in the compositions of EO from lavender showed differences in geographical, climatic, genotypic, and seasonal changes^11,12^. The EO composition of spike lavender is determined mainly by the genetic composition of cultivars, although the monoterpenes linalool, 1,8-cineol, and camphor are the major components, with more than 80% of the EO^13^. α-Bisabolene, α-pinene, α-terpineol, camphor, eucalyptol and linalool were the most abundant components for *L. latifolia* EO^14^.

Plant-based biostimulants (PDBs) have free amino acids and polypeptides that are extracted and/or hydrolyzed by enzymes. Natural biostimulant compounds are classified depending on their source and contents (humic substances, seaweed extracts, plant extracts, and products containing amino acids)^15–20^. Furthermore, PDBs can accelerate plant growth, protect plants against abiotic stresses, and improve nutrient use efficiency by improving plant physiological processes such as nutrient uptake, growth, and tolerance to abiotic stresses^21–24^.

The benefits of PDBs on growth and production, as well as the various physiological and biochemical properties of various horticultural crops, are demonstrated by several examples. For example, carrot root extract increased the growth, physiological processes, and phytochemical concentrations of cowpea seedlings under salt stress^25^. Leaf extracts from aloe, garlic, and Lawsonia as soil drench or foliar sprays improved the growth and chemical concentration of the dwarf umbrella tree (*Schefflera arboricola*)^26^. Aqueous garlic extract improved the growth, physiological traits, phytochemical content, and composition of several horticultural crops, including eggplants^27^. Comparing results to the control plants, the morphological characteristics, yield, and quality increased parallel with an increase in the amounts of organic and vermicompost fertilizer in *Nigella damascena* plant^28^.

Humic substances, such as humic acids (HAs), are major organic matter in soils produced and accumulated mainly through the biochemical decomposition of plant and animal residues by microbial synthetic activity^29–33^. The application of humic substances to medicinal and aromatic plants has gained prominence recently. HAs can improve the physicochemical properties of soils by increasing their organic matter contents and microbial populations^34–36^.

Sustainable and environmentally friendly agricultural practices have become increasingly important in global agriculture due to the growing demand for natural therapeutic plant products^37,38^. Due to agricultural needs, there is a constant search for environmentally acceptable and sustainable alternatives.

Therefore, we must increase the efficiency of nutrient uptake and strengthen plants’ natural defense mechanisms against biotic and abiotic stresses to ensure sustainable natural agroecosystems for future generations without increasing the use of fertilizers and pesticides, while also improving crop safety and yield^39,40^. Research on natural resources, like plant extracts, is being investigated as potential substitutes for conventional chemicals in light of these goals. These natural substances or bioactive phytocompounds that act as biostimulants can promote plant growth, lessen the impact of biotic and abiotic stressors, and lessen the growing system’s reliance on pesticides and fertilizers^41–44^. Additionally, the rhizosphere soil microbiome can be considerably altered by the administration of plant extracts by soil soaking, which can also indirectly interact with plants to stabilize plant growth^45^. Furthermore, plant extracts can be derived from abundant raw materials, such as weeds, wild plants, and other non-economic plants.

Mallaw plant (*Malva parviflora* L.) is a perennial herbaceous plant from the Malvaceae family, largely distributed in areas with tropical, subtropical, and moderate climates in Asia, Africa, and Europe^46^ and it spreads as a wild plant in Egypt. Medicinal plants have several vital chemical compounds and some biological functions^47^. Its high nutritional content comes from the presence of salicylic acid, vitamins A, C, B3, B2, B1, and E, and helpful minerals like calcium, magnesium, phosphorus, potassium, copper, iron and zinc^46,48^. The leaves of plants are a good source of some phenolic compounds and anti-oxidant compounds and have also been found to be effective against bacteria and fungi infections^49–51^.

The mallow is a nutrient-rich, wild-growing plant in Egypt. Therefore, to optimize the plant’s benefits, this study aims to take advantage of the impact of humic acid and mallow extract on the lavender plant’s growth and oil production. It was investigated under prevailing conditions in Alexandria, Egypt.

## Materials and methods

### Field experiment

*Lavandula latifolia* Medik. plants were arranged in a split-plot model with two treatments performed in the randomized complete block design (RCBD) with three replications at two successive seasons (2022 and 2023) at the nursery of the Department of Floriculture, Ornamental Horticulture, and Garden Design, Faculty of Agriculture, Alexandria University, Egypt. Spike lavender stem cuttings were planted in March, and then the rooted cuttings were transplanted after one month for both seasons (2022 and 2023), where four similar plants for each treatment (plot) were chosen.

### Treatments

The first factor was humic acid (HA) fertilizer (Table 1), which added at four concentrations of 0 (as a control), 1, 2, and 4 g/L to the soil (Table 2). The second factor was the application of *Malva parviflora* L. (mallow) leaf aqueous extract at concentrations of 0 (as a control), 2, 4, and 6 g/L as a foliar application every two weeks for four months during the two experimental seasons. Tables 1 and 2 show the chemical analysis of HA and the soil.

**Table 1:** Chemical analysis of humic acid.

Element	Value
pH	10.43
EC (ds/m)	2.79
K %	6.50
K_2_O %	7.80
Humic %	76.00

**Table 2:** Chemical analysis of soil.

Element	Value
pH	7.61
EC (ds/m)	3.80
Soluble cations (meq/L)	
Na^+^	13.30
K^+^	3.80
Ca^+ 2^	11.00
Mg^+ 2^	9.00
Soluble anions (meq/L)	
Cl^−^	10.50
HCO^3−^	12.00
CO3^−2^	0.00
SO4^−2^	15.50

### Collection and preparation of *M. Parviflora* plant

*M. parviflora* plants were collected in March 2022 and 2023 at the flowering stage from the nursery of the Department of Floriculture, Ornamental Horticulture, and Garden Design, Faculty of Agriculture, Alexandria University, Egypt. *M. parviflora* was identified by Dr. Mona Hemaid, Crop Field Department, Faculty of Agriculture, Alexandria University, Egypt. *M. parviflora* leaves were washed with tap water, dried at room temperature, and ground to a fine powder using a grinder. The material powder was kept in dry conditions until use.

#### Extraction of *M. parviflora* leaves

*M. parviflora* leaf powder (100 g) was extracted by soaking it in 1000 mL of distilled water in a glass bottle at room temperature for 24 h. Then it was filtered through a muslin textile and dried in an oven at 50 °C for 72 h^52^. The final dried extract was stored at 4 °C in a refrigerator until used and analyzed by HPLC^51^. The extract percentage was calculated as follows: Extract percent = [extract amount (g)/dry leaf sample amount (g)] × 100. The extract percentage was 2.21%.

#### Water-soluble vitamins

Water soluble vitamins in *M. parviflora* leaf extract were analyzed and performed by HPLC (Agilent 1100), which is composed of two LC- pumps, and a UV/Vis detector. Chromatograms were obtained and analyzed using the Agilent ChemStation. Chromatographic separation was achieved on column (Agilent ZORBAX C18; 250 × 4.6 mm i.d., with 5 μm particle size) through the isocratic delivery mobile phase (A/B 33/67; A: MeOH, B: 0.023 M H_3_PO_4_, pH = 3.54) at a flow rate of 1.0 mL/min. The UV absorbance was recorded at 270 nm at room temperature^53,54^.

#### Analysis of phenolic and flavonoid compounds by HPLC

Analyses of phenolic and flavonoid compounds were performed using HPLC apparatus (Agilent Series 1100) (Agilent, USA) composed of auto-sampling injector, solvent degasser, two LC-pumps (series 1100), with ChemStation software, and UV/Vis detector (set at 250 nm for phenolic acids and 360 nm for flavonoids)^55,56^. The analysis was achieved C18 column (125 mm × 4.60 mm, with 5 μm particle size). Phenolic acids were separated by employing a gradient mobile phase of two solvents- Solvent A (Methanol) and Solvent B (Acetic acid in water (1:25). The gradient program began with 100% B and was held at this concentration for the first 3 min. This was followed by 50% eluent A for the next 5 min after which the concentration of A was increased to 80% for the next 2 min and then reduced to 50% again for the following 5 min detection wavelength at 250 nm. Flavonoids were separated by employing a mobile phase of two solvents:- acetonitrile (A) and 0.2% (v/v) aqueous formic acid (B) with an isocratic elution (70:30) program. The solvent flow rate was 1 mL/min, and separation was performed at 25 °C. The injection volumes were 25 µL.

#### Preparation and identification of protein amino acids

The hydrolyzed protein amino acids were determined as represented by Passmore^57^. A sample of 5.0 mL was concentrated at 50 °C under vacuum using a rotary evaporator and dried using a freeze-drying system. Powder of *M. parviflora* extract (0.1 g) was digested with 10 mL of 6 N HCl in a sealing tube. The mixture was hydrolyzed at 110 °C for 24 h, then filtered and the hydrolyzed protein-amino acids were obtained by evaporation of the hydrolyzate till dryness. The residue was washed with DW. The volume of the filtrate was adjusted to 100 mL using DW. The sample was filtered through a 0.45 micropore filter and injected at 50 µL in the amino acid analyzer.

Sykam Amino Acid Analyzer (Sykam GmbH, Germany) with Column (LCA KO6/Na-24050313; 4.6 × 150 mm) equipped with Solvent Delivery System S 2100 (quaternary pump with flow rate: 0.45 mL/min and maximum pressure up to 400 bar; and Temperature: Gradient 57–74 °C), Autosampler S 5200, Amino Acid Reaction Module S4300 (with built-in dual filter photometer between 440 and 570 nm with constant signal output and signal summary option), and Refrigerated Reagent Organizer S 4130 was used.

### Essential oil extraction

The hydro-distillation method extracted the essential oils (EOs) using the Clevenger apparatus. About 100 g of fresh leaves were put in a 2 L flask containing 1500 mL of distilled water for 2 h, then the EOs were collected and kept at 4 °C until analysis by GC-MS as described by^58^.

### Gas chromatography-mass spectrometry (GC-MS) analysis

The chemical composition of EO was performed using a Trace GC Ultra-ISQ mass spectrometer (Thermo Scientific, Austin, TX, USA) with a direct capillary column TG–5MS (30 m × 0.25 mm × 0.25 μm film thickness). The column oven temperature was initially held at 70 °C and then increased by 5 °C/min to 280 °C withheld for 5 min then increased to 300 at 5 °C/min. The injector and MS transfer line temperatures were kept at 250 °C. Helium was used as a carrier gas at a constant flow rate of 1 mL/min. The solvent delay was 2 min and diluted samples of 1 µL were injected automatically using Autosampler AS1310 coupled with GC in the split mode. EI mass spectra were collected at 70 eV ionization voltages over the range of m/z 40–600 in full scan mode. The ion source was set at 200 °C.

The components were identified by comparison of their mass spectra with those of WILEY 09 and NIST 14 mass spectral database^59^. Xcalibur 3.0 data system in the GC–MS with its threshold values was used to confirm that all the mass spectra of the identified compounds were attached to the library. Furthermore, the measurement match factor (MF) with values ≥ 650 was used to confirm the identified compounds^58^.

### Chlorophyll content

Leaf total chlorophyll content was determined in fresh leaf samples of each plant for the different treatments at the end of the experiment as a SPAD unit^60^ using Minolta SPAD Chlorophyll meter model No. 502.

### Statistical analysis

The experiment was conducted in a complete randomized block design containing 16 treatments with three replicates in a split-plot model. The main plot and subplot factors were humic acid and *Malva parviflora* leaf extract, respectively. The collected data from both seasons were subjected to analysis of variance (ANOVA) using the SAS program, and the comparison among means was measured using LSD at the 0.05 level of probability.

## Results

### Water soluble vitamins of *Malva parviflora* leaves extract

**Fig. 1:**
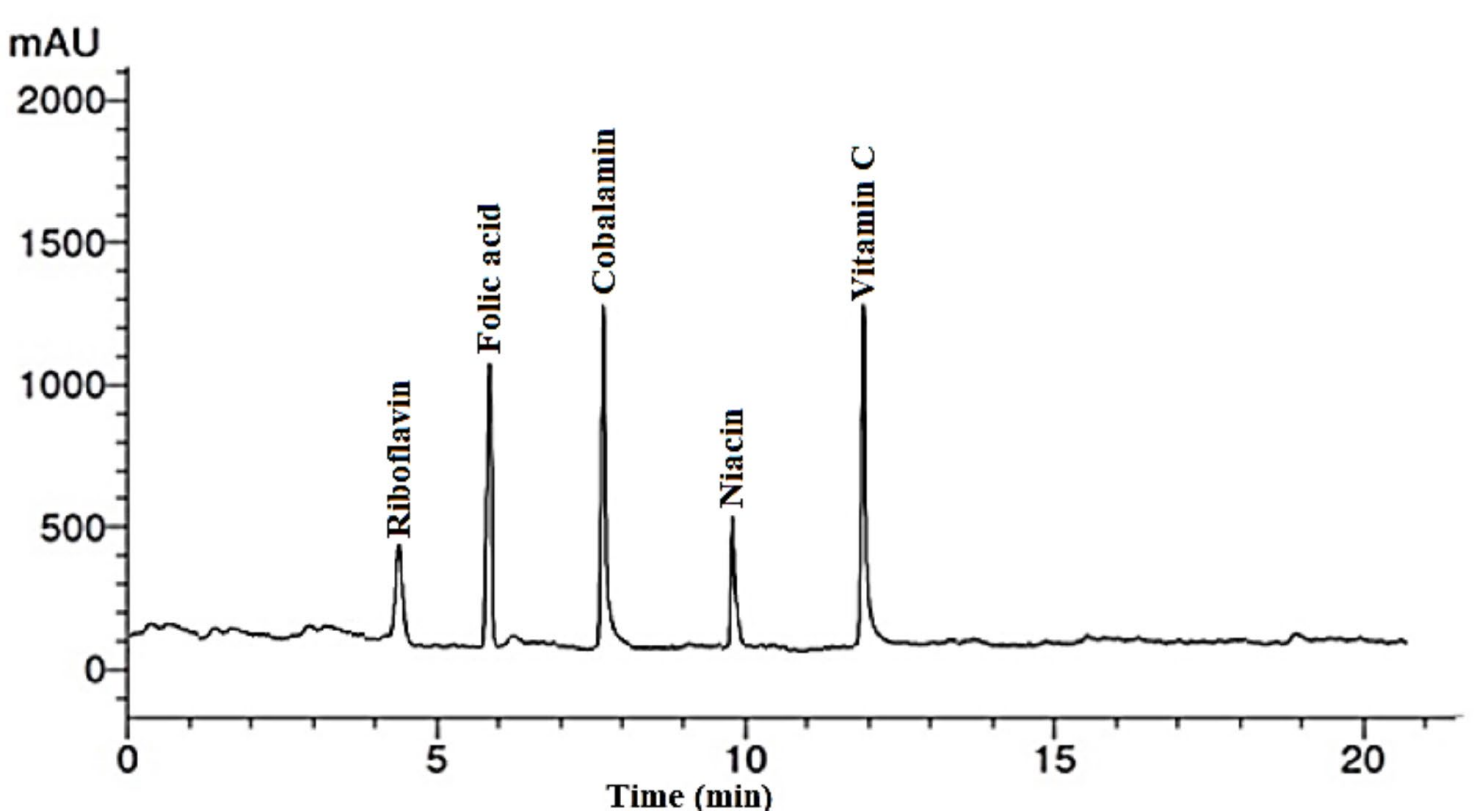
HPLC chromatograms of the identified water-soluble vitamins in *Malva parviflora* leaf extract.

Table 3 presents the vitamin compounds identified in the water extract of *Malva parviflora* leaves (ME), and the separation chromatograms by HPLC are shown in Fig. 1. The results indicated that the most abundant vitamins were vitamin B12 (cobalamin) (15.42 µg/g extract), vitamin C (14.10 µg/g extract), and folic acid (10.35 µg/g extract).

**Table 3:** Concentrations of water-soluble vitamins in *Malva parviflora* leaf extract.

RT (min)	Compound	Concentration (µg/g extract)
4.2	Vit.B2 (Riboflavin)	2.49
6.0	Folic acid	10.35
8.0	Vit.B12 (Cobalamin)	15.42
10.0	Niacin (nicotinic acid)	5.28
12.0	Vitamin C	14.10

### Phenolic and flavonoid compounds of *Malva parviflora* leaf extract

**Fig. 2:**
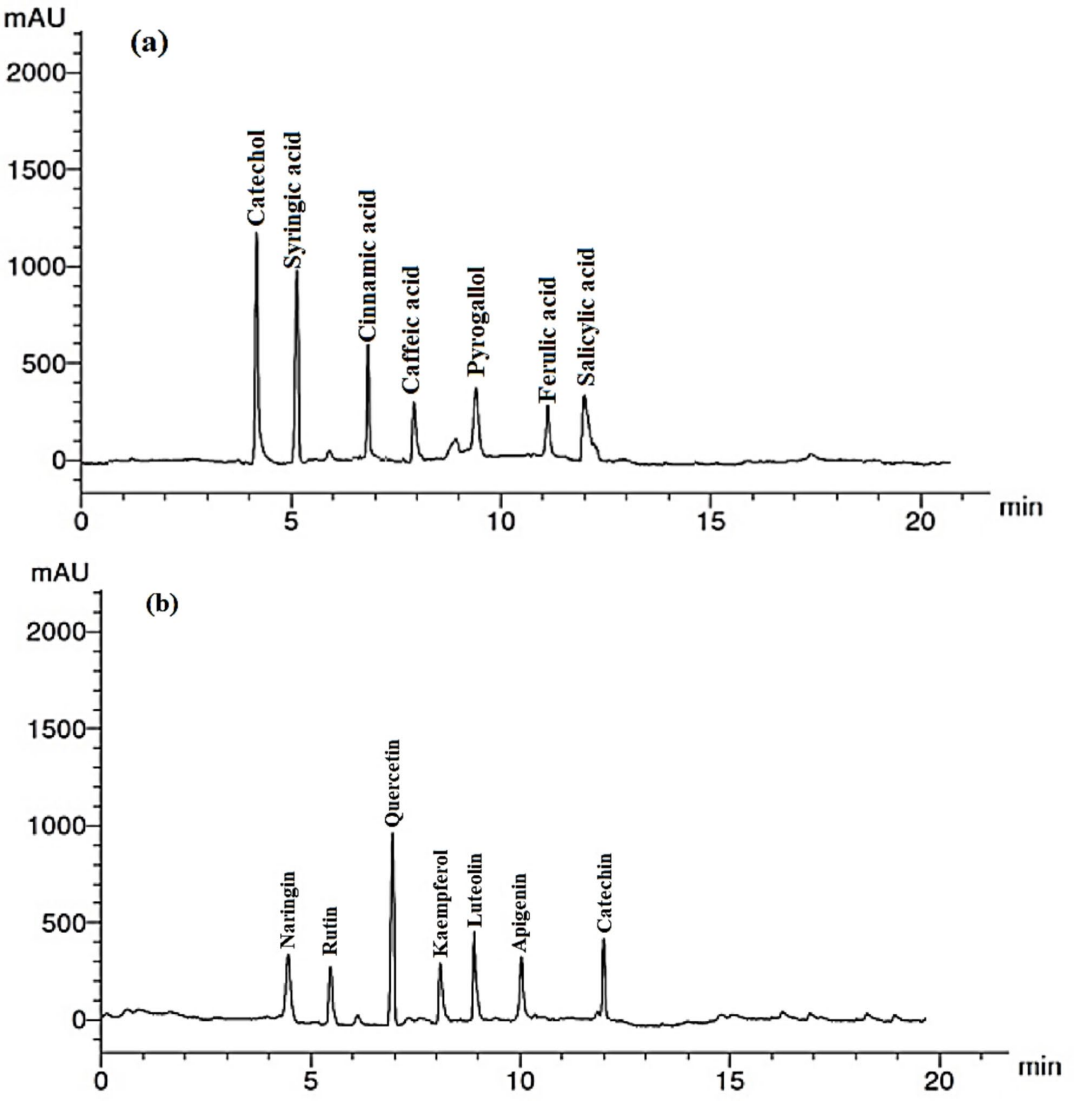
The HPLC analysis of the phenolic and flavonoid compounds in *Malva parviflora* leaf extract.

The phenolic and flavonoid compounds identified in *M. parviflora* leaf extract are listed in Table 4, and the HPLC analysis for phenolic and flavonoid compounds is shown in Fig. 2a and b, respectively. The results showed that the abundant phenolic compounds were catechol (11.22 µg/mL extract), syringic acid (9.22 µg/mL extract), and cinnamic acid (6.16 µg/mL extract), while the abundant flavonoid compounds were quercetin (11.22 µg/mL extract), luteolin (5.06 µg/mL extract), and catechin (4.07 µg/mL extract).

**Table 4:** The phenolic and flavonoid compounds in *Malva parviflora* leaf extract.

RT* (min)	Phenolic compound	Concentration (µg/mL extract)	RT (min)	Flavonoid compound	Concentration (µg/mL extract)
4.0	Catechol	11.22	4.6	Naringin	3.14
5.0	Syringic acid	9.22	5.2	Rutin	2.90
7.0	Cinnamic acid	6.16	6.9	Quercetin	11.22
8.0	Caffeic acid	3.34	8.1	Kaempferol	2.46
9.4	Pyrogallol	3.14	9.0	Luteolin	5.06
11.0	Ferulic acid	2.44	10.0	Apigenin	3.38
12.0	Salicylic acid	3.98	12.01	Catechin	4.07

RT, retention time (min).

#### Compounds of amino acids of *Malva parviflora* leaf extract

**Fig. 3:**
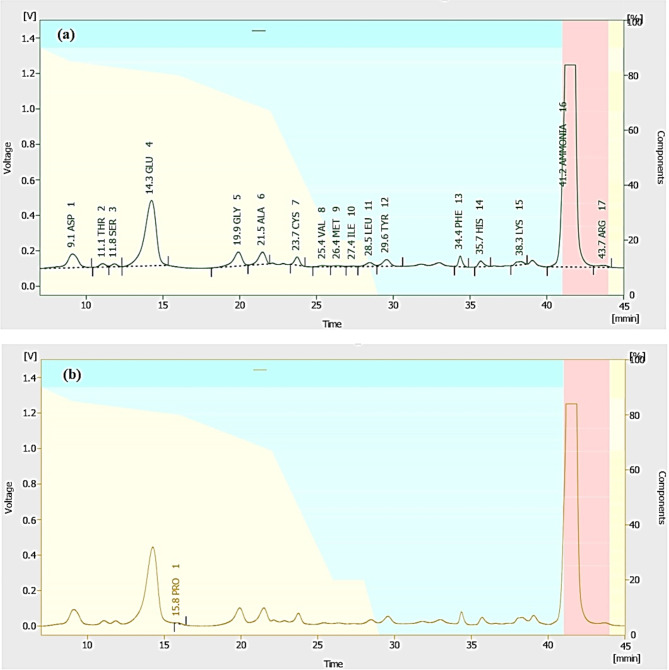
Chromatograms of the identified amino acids of *Malva parviflora* leaf extract (a) and proline (b).

As shown in Table 5, the amino acid analyzer revealed that ME contained several nitrogenous compounds and amino acids. The most abundant compounds were glutamic acid (19.38 mg/g), ammonia (8.52 mg/g), and aspartic acid (3.34 mg/g), followed by alanine (1.47 mg/g), glycine (1.35 mg/g), and tyrosine (1.23 mg/g). Figure 3a shows the HPLC chromatograms of nitrogenous compounds and amino acids of ME. Figure 3b shows the HPLC chromatogram of proline compound.

**Table 5:** Amino acids contents of *Malva parviflora* leaf extract.

Retention time (min)	Compound name	Concentration (mg/g)
9.109	ASP (Aspartic acid)	3.34
11.077	THR (Threonine)	0.40
11.840	SER (Serine)	0.31
14.259	GLU (Glutamic acid)	19.38
15.832	PRO (Proline)	0.09
19.917	GLY (Glycine)	1.35
21.485	ALA (Alanine)	1.47
23.739	CYS (Cysteine)	0.92
25.411	VAL (Valine)	0.15
26.363	MET (Methionine)	0.17
27.355	ILE (Isoleucine)	0.09
28.469	LEU (Leucine)	0.51
29.568	TYR (Tyrosine)	1.23
34.357	PHE (Phenylalanine)	0.86
35.699	HIS (Histidine)	0.56
38.301	LYS (Lysine)	0.60
41.181	AMM (Ammonia)	8.52
43.688	ARG (Arginine)	0.60

### Vegetative parameters, oil content and chlorophyll content

The effect of different levels of humic acid (HA) and *Malva parviflora* leaf extract (ME) on the vegetative parameters, oil content, and chlorophyll content of Spike lavender in both seasons are shown in Table 6.

Generally, the data in Table 4 indicated that using ME alone at different concentrations had not significant effect on the vegetative growth parameters of Spike lavender (plant height, branch number, plant fresh weight, and leaf area), compared with control treatment, with some exceptions when using a high concentration of ME.

Moreover, applying HA alone, especially at 2–4 g/L caused significant increases in all vegetative growth parameters of Spike lavender, compared with untreated plants. Furthermore, the combination of HA at 4 g/L and ME at 2–4 g/L gave the maximum significant values of vegetative growth parameters, compared with the other treatments.

The height of the plant was affected significantly (*p* < 0.05) by the treatments, and the values ranged from 30.50 to 43.66 cm and from 34.65 to 42.51 cm in the first and second seasons, respectively. In the first season, the treated plants with HA and ME at dosages of 4 + 6 g/L, 4 + 2 g/L, and 4 + 0 g/L showed the highest plant height values of 43.66, 40.41, and 38.25 cm, respectively. In the second season, the treated plants by HA and ME at dosages of 2 + 4 g/L, 4 + 6 g/L, 4 + 4 g/L, 1 + 4 g/L, and 2 + 6 g/L observed the highest plant height values of 42.51, 42.11, 41.75, 41.50, and 41.44 cm, respectively.

The number of branches/plant was 29.50–49.00 and 28.14–47.33 branches per plant in the first and second seasons, respectively. The highest number of branches/plant were found to be 49.00, 48.16, 45.66, 44.16, 43.66, and 43.33 in the plants treated with HA and ME at concentrations of 4 + 2 g/L, 4 + 0 g/L, 4 + 6 g/L, 2 + 6 g/L, 2 + 0 g/L, and 2 + 4 g/L, respectively, in the first season. In the second season, the number of branches/plant reached the highest values of 47.33, 47.22, 46.27, 43.88, 43.80, 42.50, and 42.44, as the plants were treated with HA and ME at concentrations of 4 + 6 g/L, 4 + 4 g/L, 4 + 2 g/L, 1 + 6 g/L, 2 + 6 g/L, 2 + 4 g/L, and 1 + 2 g/L, respectively.

Leaf area values ranged from 1.39 to 2.16 cm^2^ and from 1.31 to 2.84 cm^2^ in the first and second seasons, respectively. In the first season, the highest leaf area values (2.16, 2.12, 2.11, 2.06, and 2.05 cm^2^) were observed as the plants treated with HA and ME at concentrations of 4 + 4 g/L, 4 + 0 g/L, 4 + 6 g/L, 2 + 2, and 4 + 2 g/L, respectively. In the second season, the plants treated with 4 + 4 g/L, 4 + 2 g/L, 2 + 4 g/L, 4 + 0 g/L, 2 + 6 g/L, 1 + 6 g/L, 2 + 0 g/L, and 0 + 6 g/L showed the highest leaf area values of 2.84, 2.71, 2.68, 2.59, 2.51, 2.19, 2.07, and 2.03 cm^2^, respectively.

According to the data of the total fresh weight (TFW g), the values ranged from 34.37 to 50.08 g and from 29.52 to 47.02 g in the first and second seasons, respectively. In the first season, the highest TFW values of 50.08, 48.51, 47.35, and 44.34 g were obtained from the treated plants with the concentrations of HA and ME at 4 + 2 g/L, 4 + 6 g/L, 4 + 0 g/L, and 2 + 2 g/L, respectively, compared with untreated plants (35.31 g). In the second season, the plants treated with HA and ME at concentrations of 4 + 6 g/L, 4 + 2 g/L, 4 + 4 g/L, and 2 + 4 g/L showed the highest TFW values of 45.83, 47.02, 44.53, and 44.07 g, respectively, compared with the control (32.61 g).

According to data in Table 6, HA at different concentrations with or without ME had a significant effect on the EO (%), which ranged from 0.37 to 0.62% in the first season, compared with control (0.30%) and from 0.54 to 0.84%, compared with control (0.48%) in the second season. The highest EO % was obtained by treatments HA and ME at 4 + 0 g/L, 2 + 0 g/L, 4 + 4 g/L, 4 + 2 g/L, and 4 + 6 g/L in the first season, with values of 0.62, 0.61, 0.58, 0.57, and 0.55%, respectively. In the second season, the plants treated with HA and ME at 4 + 6 g/L, 2 + 0 g/L, 4 + 0 g/L, 4 + 0 g/L, and 2 + 6 g/L showed the highest values of EO (%) of 0.84, 0.82, 0.79, and 0.78%, respectively.

For the chlorophyll content, the treated plants with HA and ME had a significant effect compared with untreated plants, where the plants treated with HA and ME at 4 + 4 g/L had the highest chlorophyll content (57.07 SPAD Unit), followed by 2 + 2 g/L (56.21 SPAD Unit) compared with control plants (44.25 SPAD Unit) in the first season, while the treatments 4 + 4 g/L and 4 + 6 g/L showed the highest values of 58.65 and 58.38 SPAD Unit, respectively, compared with control plants (45.8 SPAD Unit).

**Table 6:** Vegetative parameters and biochemical composition of *Lavandula latifolia* plants as affected by the treatments of humic acid and *Malva parviflora* extract in two seasons 2022–2023.

Treatment		Plant height (cm)		No. branches/plant		Leaf area (cm^2^)		Total fresh weight (g)		Essential oil (%)		Chlorophyll content (SPAD unit)	
HA g/L	ME g/L	1st	2nd	1st	2nd	1st	2nd	1st	2nd	1st	2nd	1st ns	2nd
0	0	30.50 ± 0.87^*i*^	35.15 ± 2.01^*d*^	29.50 ± 0.1.5^*j*^	30.16 ± 3.01^*f*^	1.39 ± 0.35^*e*^	1.31 ± 0.40^*f*^	35.31 ± 1.18 ^*gh*^	32.61 ± 3.28^*de*^	0.30 ± 0.05^ef^	0.48 ± 0.017^gh^	44.25 ± 3.03^f^	45.80 ± 1.00^g^
	2	32.33 ± 0.58^*ghi*^	36.00 ± 1.32^*cd*^	30.33 ± 2.75^*ij*^	28.14 ± 3.16^*f*^	1.55 ± 0.17^*c-e*^	1.50 ± 0.09^*ef*^	34.37 ± 2.65^*h*^	29.52 ± 3.01^*e*^	0.35 ± 0.03^d-f^	0.41 ± 0.04^h^	44.49 ± 0.96^f^	44.45 ± 2.52^g^
	4	30.83 ± 2.93^*i*^	34.65 ± 1.38^*d*^	34.83 ± 2.75^*f-h*^	31.11 ± 1.2^*ef*^	1.85 ± 0.29^*a-d*^	1.84 ± 0.18^*de*^	37.16 ± 2.95^*e-h*^	33.87 ± 2.3^*de*^	0.27 ± 0.07^f^	0.51 ± 0.02^f-h^	50.14 ± 1.22^e^	50.17 ± 3.65^f^
	6	33.33 ± 1.18^*fghi*^	36.36 ± 1.38^*cd*^	36.00 ± 2.18^*f-h*^	36.03 ± 2.42^*de*^	1.66 ± 0.039^*b-e*^	2.03 ± 0.26^*d*^	42.58 ± 0.63^*c-e*^	32.65 ± 0.62^*de*^	0.44 ± 0.08^b-f^	0.57 ± 0.01^e-g^	51.6 ± 1.38^de^	52.65 ± 2.25^ef^
1	0	31.91 ± 1.28^*g-i*^	37.88 ± 3.72^*b − d*^	32.50 ± 1.73^*hij*^	35.94 ± 4.00^*de*^	1.40 ± 0.15^*de*^	1.92 ± 0.26^*de*^	36.99 ± 2.03^*f-h*^	35.33 ± 2.36^d^	0.37 ± 0.085^*c-f*^	0.54 ± 0.08^e − g^	52.59 ± 2.01^c − e^	52.87 ± 0.18^ef^
	2	31.08 ± 5.15^*hi*^	40.44 ± 1.00^*ab*^	34.50 ± 3.77^*ghi*^	42.44 ± 1.92^*ab*^c	1.93 ± 0.17^*a-c*^	1.81 ± 0.07^*de*^	41.74 ± 3.52^*d-f*^	35.79 ± 3.41^*cd*^	0.45 ± 0.16^*a-f*^	0.62 ± 0.07^d − f^	51.57 ± 1.43^de^	53.17 ± 2.30^d − f^
	4	31.08 ± 2.504^*hi*^	41.50 ± 4.28^*ab*^	33.83 ± 2.75^*g-j*^	40.11 ± 4.55^*b − d*^	1.70 ± 0.06^*b-e*^	1.78 ± 0.17^*b − e*^	36.73 ± 5.2^*f-h*^	34.72 ± 3.39^*d*^	0.43 ± 0.03^*b-f*^	0.62 ± 0.07^d − f^	52.98 ± 1.29^b − e^	55.65 ± 2.77^a − e^
	6	37.66 ± 3.05b^*b-d*^	40.50 ± 1.59^*ab*^	37.33 ± 2.50^*efg*^	43.88 ± 0.19^*ab*^	1.89 ± 0.11^*a-c*^	2.19 ± 0.14^*b − d*^	43.47 ± 1.65^*b-d*^	40.82 ± 3.10^*b*^	0.468 ± 0.08^*a-e*^	0.69 ± 0.05^cd^	55.46 ± 1.41^a − c^	57.5 ± 0.34^ab^
2	0	34.25 ± 0.25^*e-e*^	39.33 ± 0.52^*ac*^	43.66 ± 5.35^*bc*^	39.39 ± 4.01^*b − d*^	1.57 ± 0.30^*c-e*^	2.07 ± 0.04^*cd*^	40.73 ± 2.71^*d-g*^	33.86 ± 1.72^*de*^	0.61 ± 0.11^*ab*^	0.82 ± 0.10^ab^	52.66 ± 2.18^a − c^	55.03 ± 0.85^b − e^
	2	37.33 ± 1.26^*b-c*^	35.72 ± 2.66^*d*^	39.00 ± 3.61^*d-f*^	39.77 ± 1.17^*b − d*^	2.06 ± 0.43^*ab*^	1.86 ± 0.26^*de*^	44.34 ± 5.3^*b-d*^	36.23 ± 3.25^*cd*^	0.46 ± 0.17^*a-f*^	0.71 ± 0.04^b − d^	56.21 ± 0.64^ab^	57.34 ± 0.74^a − c^
	4	34.83 ± 1.61^*d-g*^	42.51 ± 3.75^*a*^	43.33 ± 2.25^*c*^	42.50 ± 3.04^*a − c*^	1.94 ± 0.41^*abc*^	2.68 ± 0.48^*a*^	43.49 ± 3.33^*b-d*^	44.07 ± 3.14^*ab*^	0.52 ± 0.11^*a-d*^	0.65 ± 0.09^de^	54.6 ± 1.19^a − d^	55.68 ± 1.65^a − e^
	6	36.33 ± 3.33^*c-f*^	41.44 ± 1.42^*ab*^	44.16 ± 1.61^*bc*^	43.80 ± 1.13^*ab*^	1.84 ± 0.33^*a-e*^	2.51 ± 5.6^*a − c*^	43.67 ± 2.85^*b-d*^	43.17 ± 2.89^*ab*^	0.52 ± 0.05^*a-d*^	0.78 ± 0.16^a − c^	55.2 ± 1.61^a − c^	56.33 ± 1.27^a − d^
4	0	38.25 ± 1.95^*bc*^	39.08 ± 1.01^*a − c*^	48.16 ± 1.89^*ab*^	38.11 ± 8.37^*cd*^	2.12 ± 0.25^*ab*^	2.59 ± 0.18^*ab*^	47.35 ± 5.85^*a-c*^	40.13 ± 2.91^*bc*^	0.62 ± 0.07^a^	0.792 ± 0.1^a − c^	54.62 ± 1.17^a − d^	54.06 ± 1.55^c − e^
	2	40.41 ± 2.32^*b-d*^	40.69 ± 3.21^*ab*^	49.00 ± 1.53^*a*^	46.27 ± 3.92^*a*^	2.05 ± 0.06^*ab*^	2.71 ± 0.41^*a*^	50.08 ± 1.43^*a*^	47.02 ± 2.97^*a*^	0.57 ± 0.16^*ab*^	0.78 ± 0.06^a − c^	54.42 ± 2.17^a − d^	57.2 ± 2.90^a − c^
	4	37.5 ± 0.87^*b-d*^	41.75 ± 3.03^*a*^	41.50 ± 1.33^*c-e*^	47.22 ± 2.55^*a*^	2.16 ± 0.24^*a*^	2.84 ± 0.12 ^*a*^	41.34 ± 3.64^*d-f*^	44.53 ± 2.69^*ab*^	0.58 ± 0.06^*ab*^	0.72 ± 0.05^b − d^	57.07 ± 1.64^a^	58.65 ± 2.28^a^
	6	43.66 ± 1.04^*a*^	42.11 ± 2.12^*a*^	45.66 ± 1.9^*c-f*^	47.33 ± 2.34^*a*^	2.11 ± 0.29^*ab*^	1.99 ± 0.17 ^*d*^	48.51 ± 2.84^*ab*^	45.83 ± 3.86^*a*^	0.55 ± 0.16^*ac*^	0.84 ± 0.12^a^	55.23 ± 2.6^a − c^	58.38 ± 0.93^ab^
LSD		3.21	3.66	4.37	5.61	0.45	0.46	5.55	4.51	0.18	0.11	3.24	3.36

Values are means ± SD. Means with the same letter/s within the same column are not significantly different according to LSD at 0.05 level of probability.

### Chemical compounds of the essential oils of *Lavandula latifolia* leaves

**Fig. 4:**
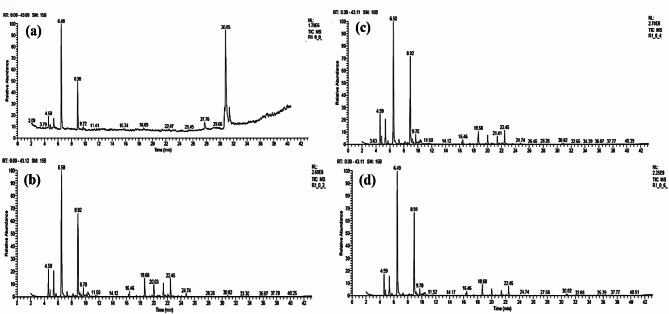
GC-MS chromatograms of the essential oils from Spike lavender. Plants treated by humic acid at 0 g/L with *Malva parviflora* extract at 0 g/L (a), 2 g/L (b), 4 g/L (c), and 6 g/L (d).

**Fig. 5:**
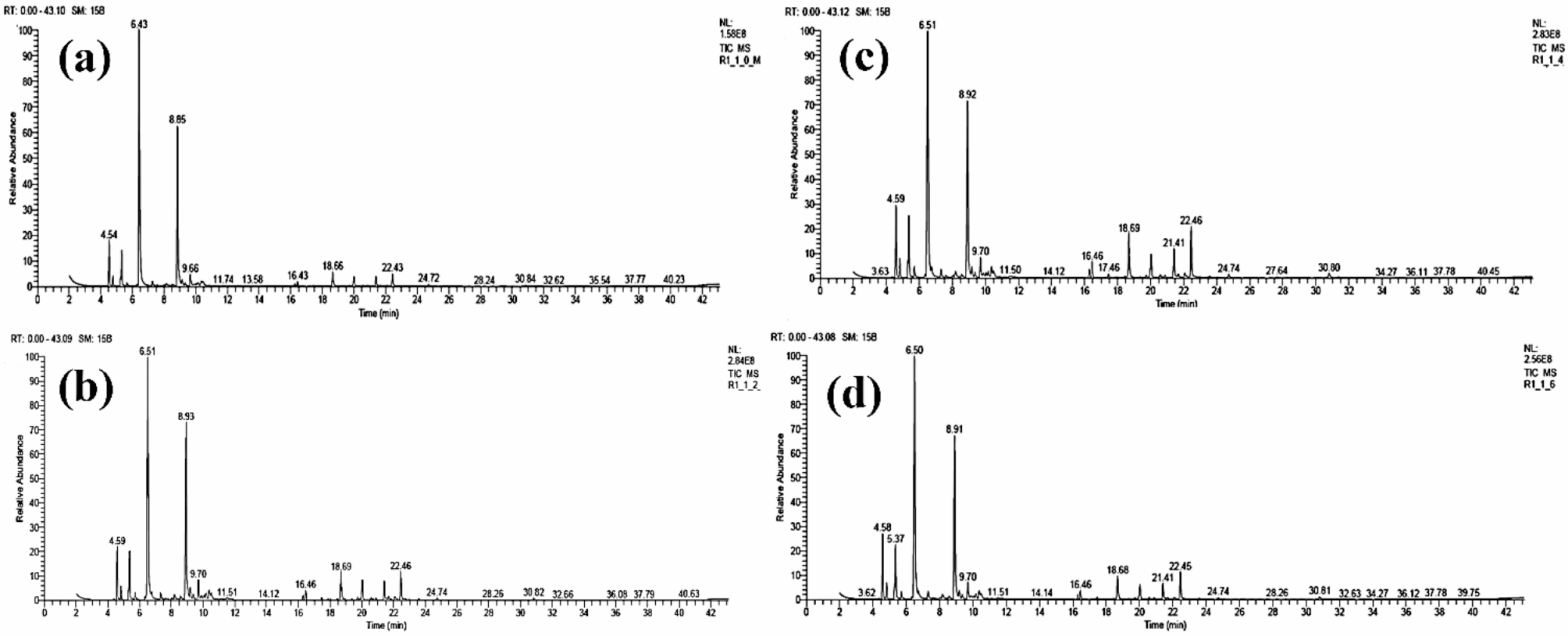
GC-MS chromatograms of the essential oils from Spike lavender. Plants treated by humic acid at 1 g/L with *Malva parviflora* extract at 0 g/L (a), 2 g/L (b), 4 g/L (c), and 6 g/L(d).

**Fig. 6:**
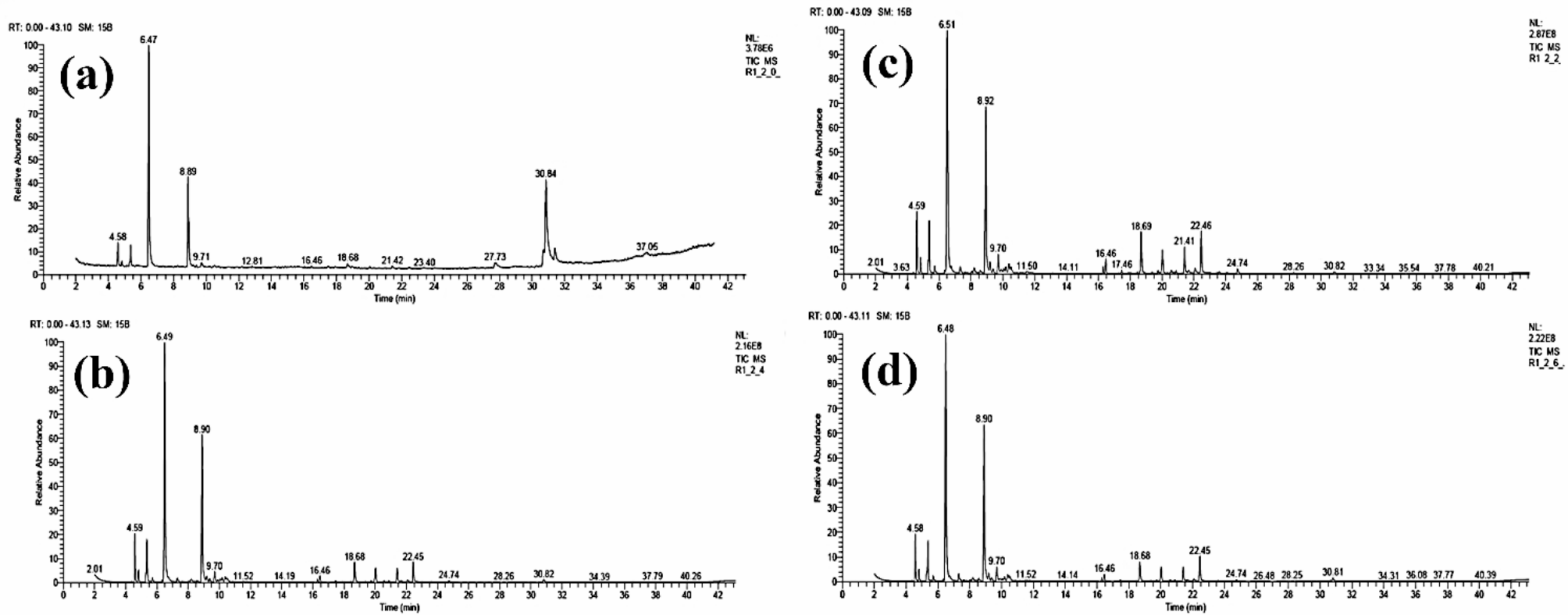
GC-MS chromatograms of the essential oils from Spike lavender. Plants treated by humic acid at 2 g/L with *Malva parviflora* extract at 0 g/L (a), 2 g/L (b), 4 g/L (c), and 6 g/L (d).

**Fig. 7:**
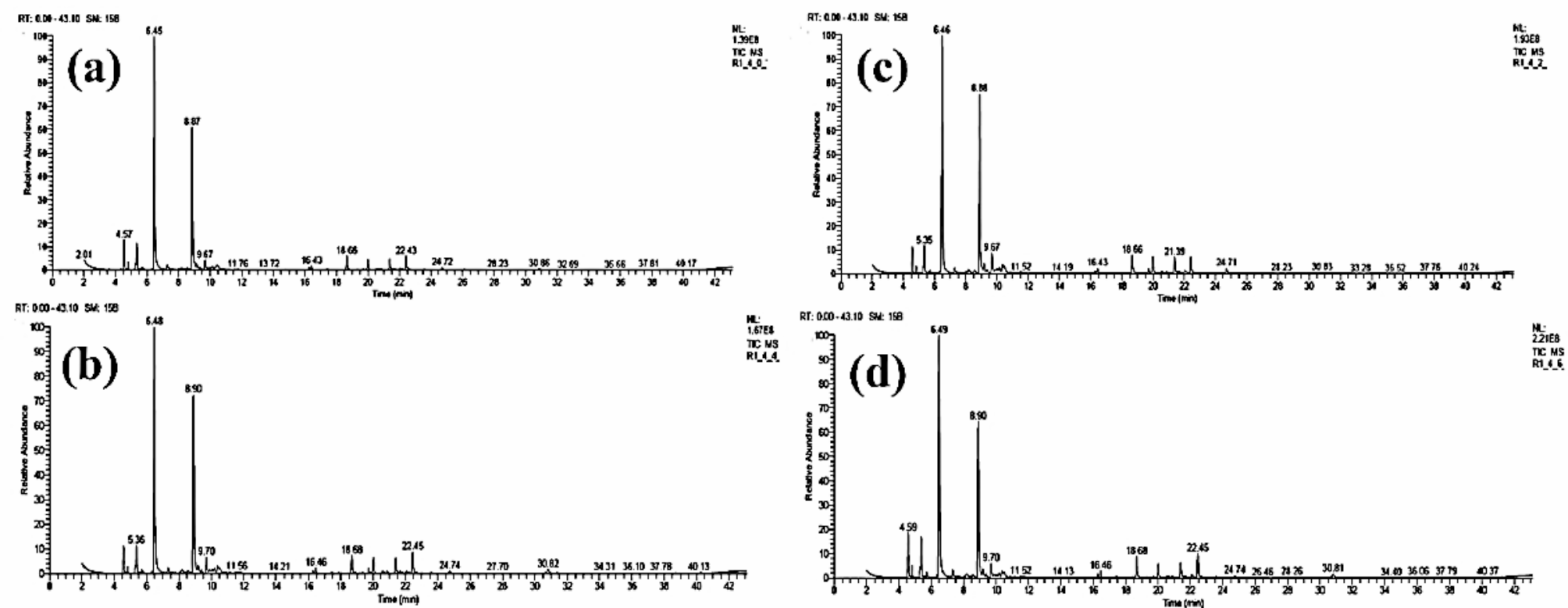
GC-MS chromatograms of the essential oils from Spike lavender. Plants treated by humic acid at 4 g/L with *Malva parviflora* extract at 0 g/L (a), 2 g/L (b), 4 g/L (c), and 6 g/L (d).

Table 7; Figs. 4, 5, 6 and 7 show the results of the chemical analysis of the essential oils (EOs) extracted from *Lavandula latifolia* leaves as affected by several treatments. Figure 4 shows the GC-MS chromatograms of the EOs from Spike lavender plants treated by HA at 0 g/L with ME at 0 g/L (Fig. 4a), 2 g/L (Fig. 4b), 4 g/L (Fig. 4c), and 6 g/L(Fig. 4d). Figure 5 shows the GC-MS chromatograms of the EOs from Spike lavender plants treated by HA at 1 g/L with ME at 0 g/L (Fig. 5a), 2 g/L (Fig. 5b), 4 g/L (Fig. 5c), and 6 g/L(Fig. 5d). Figure 6 shows the GC-MS chromatograms of the EOs from Spike lavender plants treated by HA at 2 g/L with ME at 0 g/L (Fig. 6a), 2 g/L (Fig. 6b), 4 g/L (Fig. 6c), and 6 g/L(Fig. 6d). Figure 7 shows the GC-MS chromatograms of the EOs from Spike lavender plants treated by HA at 4 g/L with ME at 0 g/L (Fig. 7a), 2 g/L (Fig. 7b), 4 g/L (Fig. 7c), and 6 g/L (Fig. 7d).

The most abundant compounds in the EOs were *α*-pinene, *β*-pinene, eucalyptol, camphor, isoborneol, *β*-fenchol, Δ-elemene, *β*-caryophyllene oxide, .tau.-cadinol, germacrene D-4-ol, and camphene, as well as oleic acid methyl ester in some treatments. These compounds were found with different concentrations in the EOs according to the treatment used, where the range of α-pinene (3.39–7.16%), camphene (0.89–1.74%), sabinene (0–4.42%), *β*-pinene (0–5.91%), eucalyptol (28.74–46.19%), camphor (15.34–30.49%), isoborneol (0–3.20%), *β*-fenchol (0–1.44%), *β*-caryophyllene (0–1.56%), Δ-elemene (0-5.08%), *β*-caryophyllene oxide (0–3.31%), *tau*.-cadinol (0–3.40%), germacrene D-4-ol (0–5.18%), oleic acid methyl ester (0–31.04%), and 17-octadecenoic acid methyl ester (0–8.00%) was reported. Treated *L. latifolia* with HA and ME affected the EO components, where some components were found in treated plants and absent in untreated plants, such as *β*-pinene, *β*-myrcene, (*E*)-α-ocimene, *cis*-thujane-4-ol, linalool, angelicoidenol, *trans*-verbenol, isoborneol, *β*-fenchol, *β*-caryophyllene, *tau*.-cadinol, Δ-elemene, and germacrene D-4-ol.

The highest percentage of *α*-pinene was obtained in the EO of treated plants with HA and ME concentrations at 1 + 6 g/L (7.16%), followed by 2 + 4 g/L (6.54%). For percentage of *β*-pinene, the most affected treatments of HA and ME were at 1 + 6 g/L (5.91%), followed by 0 + 2 g/L (5.52%), and 2 + 4 g/L (5.44%). The highest percentage value of eucalyptol in the EOs was observed in the plants treated with HA and ME concentrations at 4 + 0 g/L (46.19%), followed by 2 + 0 g/L (43.44%), compared with untreated plants (28.74%).

The highest percentage value of camphor in the EOs was found using HA and ME at 4 + 0 g/L (30.49%) and 4 + 2 g/L (30.37%), compared with control plants (15.34%). The highest percentage of isoborneol in the EOs was obtained in the plants treated by HA and ME at 4 + 2 g/L (3.20%) and was not detected in the control and 2 + 0 g/L treatments. Δ-Elemene was observed at a high percentage in the EO of plants treated with HA and ME at 0 + 2 g/L (5.08%), followed by 2 + 2 g/L (4.94%), and was not observed in the control and 2 + 0 g/L-treated plants. A high percentage of *β*-caryophyllene oxide was found in the EOs of plants treated with HA and ME at 0 + 2 g/L (3.31%), followed by 2 + 2 g/L (2.87%), and was not found in control or 2 + 0 g/L-treated plants.

For *tau*.-cadinol, the highest value was obtained using concentrations of HA and ME at 0 + 2 g/L (3.40%), followed by 2 + 2 g/L (2.95%), and absent in control and 2 + 0 g/L-treated plants. The highest percentage value of germacrene D-4-ol was obtained in the EOs of plants treated by HA and ME at 1 + 4 g/L (5.18%) and 2 + 2 g/L (4.70%). The highest percentages of oleic acid methyl ester and 17-octadecenoic acid methyl ester were observed in the EOs from untreated plants, with percentages of 31.04% and 8.00%, respectively, followed by 2 + 0 g/L (20.49 and 4.00%, respectively).

**Table 7:** Variation in the chemical compounds of the essential oils from *Lavandula latifolia* plants as affected by the treatments of humic acid and *Malva parviflora* extract.

Compound name	Percentage of compounds in the essential oils from plants treated with HA (g/L) + ME (g/L)															
	0 + 0	0 + 2	0 + 4	0 + 6	1 + 0	1 + 2	1 + 4	1 + 6	2 + 0	2 + 2	2 + 4	2 + 6	4 + 0	4 + 2	4 + 4	4 + 6
*α*-Pinene	3.55* (892)**	6.06 (937)	6.35 (950)	5.44 (959)	6.44 (947)	5.39 (952)	6.32 (945)	7.16 (946)	4.54 (919)	5.91 (946)	6.54 (944)	5.93 (948)	5.15 (940)	3.39 (946)	3.53 (945)	5.55 (948)
Camphene	1.51 (691)	1.52 (971)	1.68 (969)	1.36 (965)	1.47 (956)	1.43 (966)	1.61 (956)	1.74 (969)	1.16 (911)	1.53 (965)	1.58 (970)	1.50 (969)	1.33 (967)	0.92 (967)	0.89 (971)	1.44 (966)
Sabinene	3.11 (848)	1.22 (948)	1.14 (955)	1.01 (951)	1.04 (942)	1.06 (947)	1.27 (960)	1.35 (953)	ND	1.10 (953)	1.20 (952)	1.03 (949)	4.42 (951)	0.80 (954)	0.76 (940)	1.05 (946)
*β*-Pinene	ND	5.52 (961)	5.15 (953)	4.77 (963)	5.00 (952)	4.91 (944)	5.32 (952)	5.91 (943)	3.71 (907)	5.02 (947)	5.44 (945)	4.83 (948)	ND	3.53 (949)	3.74 (955)	4.93 (962)
*β*-Myrcene	ND	ND	0.78 (952)	0.70 (942)	0.54 (939)	0.82 (951)	1.02 (961)	0.95 (952)	ND	0.82 (951)	0.74 (933)	0.73 (953)	ND	0.41 (919)	0.50 (929)	0.77 (938)
*o*-Cymene	ND	ND	ND	ND	ND	0.31 (941)	0.27 (941)	ND	ND	0.30 (940)	ND	ND	ND	ND	ND	ND
Eucalyptol	28.74 (948)	37.69 (926)	35.74 (927)	40.01 (936)	42.30 (932)	35.05 (925)	31.68 (925)	36.57 (927)	43.44 (957)	32.70 (932)	40.33 (931)	39.18 (933)	46.19 (933)	36.93 (928)	39.80 (931)	39.20 (929)
(*E*)-α-Ocimene	ND	ND	0.36 (909)	ND	ND	0.35 (894)	0.56 (927)	0.46 (921)	ND	0.31 (915)	ND	ND	ND	ND	ND	ND
*cis*-Thujane-4-ol	ND	1.47 (954)	1.36 (952)	0.81 (953)	0.82 (940)	0.85 (954)	0.91 (951)	1.12 (952)	ND	0.86 (948)	0.71 (944)	1.25 (952)	ND	0.83 (949)	0.95 (944)	1.22 (945)
Linalool	ND	ND	0.72 (916)	ND	ND	0.68 (920)	0.67 (932)	0.64 (915)	ND	0.65 (923)	ND	0.59 (895)	ND	0.66 (926)	ND	0.55 (903)
Angelicoidenol	ND	ND	0.57 (827)	ND	ND	0.58 (814)	0.45 (843)	0.49 (832)	ND	0.48 (821)	ND	ND	ND	ND	ND	ND
Camphor	15.34 (951)	24.91 (948)	25.90 (948)	27.85 (952)	28.24 (947)	25.68 (946)	21.72 (948)	24.38 (943)	20.26 (955)	22.26 (946)	25.42 (948)	25.64 (948)	30.49 (950)	30.37 (950)	28.87 (951)	25.79 (945)
*trans*-Verbenol	ND	ND	1.16 (942)	1.02 (930)	0.91 (920)	1.24 (945)	0.97 (935)	0.98 (939)	ND	1.12 (945)	0.85 (933)	1.05 (936)	ND	1.26 (939)	1.05 (927)	1.10 (941)
Pinocarvone	ND	ND	0.57 (947)	0.55 (897)	ND	0.58 (942)	0.49 (942)	0.53 (901)	ND	0.47 (896)	0.52 (900)	0.50 (887)	ND	0.52 (935)	0.49 (923)	0.50 (898)
Isoborneol	ND	2.48 (901)	2.48 (901)	2.22 (900)	2.10 (903)	2.48 (899)	2.15 (904)	2.27 (906)	ND	2.22 (904)	1.84 (900)	2.27 (897)	2.14 (912)	3.20 (907)	2.71 (914)	2.18 (902)
Terpinen-4-ol	ND	ND	ND	ND	ND	0.45 (884)	0.40 (875)	ND	ND	0.45 (886)	ND	ND	ND	ND	ND	ND
Myrtenal	ND	ND	0.66 (906)	0.64 (885)	ND	0.70 (910)	0.56 (903)	0.59 (904)	ND	0.64 (903)	0.62 (899)	0.55 (898)	ND	0.66 (900)	0.63 (882)	0.58 (898)
*β*-Fenchol	ND	1.38 (932)	1.18 (936)	1.03 (931)	0.89 (904)	1.26 (932)	1.28 (938)	1.24 (947)	ND	1.14 (932)	0.91 (918)	1.01 (923)	ND	1.44 (917)	1.27 (935)	1.02 (933)
3-Caren-10-al	ND	ND	0.89 (830)	0.83 (837)	0.79 (817)	0.94 (831)	0.72 (839)	0.77 (827)	ND	0.81 (837)	0.62 (822)	0.82 (826)	ND	1.05 (874)	0.92 (855)	0.83 (825)
*α*-Gurjunene	ND	ND	0.56 (954)	0.62 (951)	0.47 (926)	0.56 (957)	0.81 (954)	0.61 (955)	ND	0.77 (952)	0.50 (931)	0.52 (943)	ND	0.37 (935)	0.43 (942)	0.54 (927)
*β*-Caryophyllene	ND	1.35 (948)	1.09 (944)	1.24 (939)	0.81 (931)	1.12 (938)	1.55 (949)	1.07 (947)	ND	1.56 (954)	0.99 (944)	1.00 (931)	ND	0.76 (941)	0.86 (937)	1.01 (946)
Δ-Elemene	ND	5.08 (893)	3.35 (901)	3.35 (901)	2.63 (886)	3.50 (905)	4.76 (901)	3.21 (908)	ND	4.94 (898)	3.39 (900)	3.04 (899)	3.09 (895)	3.08 (900)	2.82 (904)	3.31 (905)
Thujopsenal	ND	ND	ND	ND	ND	0.31 (797)	0.26 (801)	ND	ND	0.33 (798)	ND	ND	ND	0.78 (828)	0.80 (819)	ND
*β*-Caryophyllene oxide	ND	3.31 (929)	2.45 (925)	2.19 (927)	1.81 (921)	2.58 (933)	2.65 (926)	2.10 (929)	ND	2.87 (930)	2.44 (935)	2.29 (925)	2.22 (924)	2.70 (925)	2.58 (924)	2.19 (927)
Ledol	ND	ND	ND	ND	ND	ND	0.30 (909)	ND	ND	0.37 (882)	ND	ND	ND	ND	ND	ND
Epicubenol	ND	ND	ND	ND	ND	ND	0.28 (909)	ND	ND	0.29 (912)	ND	ND	ND	ND	ND	ND
.*tau*.-Cadinol	ND	3.40 (936)	1.95 (932)	1.56 (930)	1.65 (927)	2.21 (934)	2.93 (937)	2.05 (937)	ND	2.95 (942)	2.20 (924)	2.07 (935)	2.17 (925)	2.46 (929)	2.32 (928)	2.17 (932)
.tau.-Muurolol	ND	ND	ND	ND	ND	0.39 (882)	0.33 (917)	ND	ND	0.33 (905)	ND	ND	ND	2.44 (922)	ND	ND
*cis*-14-nor-Muurol-5-en-4-one	ND	ND	0.48 (898)	ND	ND	0.50 (888)	0.62 (904)	ND	ND	0.73 (905)	ND	ND	ND	ND	ND	ND
Germacrene D-4-ol	ND	4.62 (924)	3.42 (926)	2.78 (912)	2.10 (919)	3.38 (925)	5.18 (935)	3.51 (924)	ND	4.70 (927)	3.16 (913)	3.62 (913)	2.81 (910)	ND	3.43 (917)	3.52 (928)
Methyl-9,9,10,10-D4-octadecanoate	3.82 (701)	ND	ND	ND	ND	ND	ND	ND	ND	ND	ND	ND	ND	ND	ND	ND
8,11-Octadecadienoic acid methyl ester	4.90 (815)	ND	ND	ND	ND	ND	ND	ND	2.40 (785)	ND	ND	ND	ND	ND	ND	ND
Oleic acid methyl ester	31.04 (919)	ND	ND	ND	ND	ND	0.52 (946)	ND	20.49 (910)	ND	ND	0.56 (936)	ND	ND	0.64 (929)	0.55 (931)
17-Octadecenoic acid methyl ester	8.00 (714)	ND	ND	ND	ND	ND	ND	ND	4.00 (728)	ND	ND	ND	ND	ND	ND	ND

*: Values are the percentage of the compounds. **MF: match factor value in bracket. All analysis of studied samples have been done using Thermo fisher GC-MS which supported by number of mass spectral libraries and all separated compounds haven been identified and all data have been reported using NIST014 for 82,868 compounds (https://www.nist.gov/system/files/documents/srd/NIST1aVer22Man.pdf).

ND, not detected.

## Discussion

In the present work, using *Malva parviflora* extract (ME) as a biostimulant in conjunction with humic acid (HA) encouraged the growth of *Lavender latifolia*. The nutrients that are present in ME could promote the growth of *L. latifolia* plants. As the water-soluble vitamins folic acid, niacin (nicotinic acid), Vit B2 (riboflavin), Vit B12 (cobalamin), and vitamin C were identified in the ME, nitrogenous compounds and amino acids, alanine, ammonia, aspartic acid, glutamic acid, glycine, and tyrosine were reported. Phenolic compounds caffeic acid, catechol, cinnamic acid, ferulic acid, pyrogallol, salicylic acid and syringic acid, as well as flavonoid compounds apigenin, catechin, kaempferol, luteolin, naringin, rutin and quercetin were identified by HPLC analysis. These compounds present in the extract could enhance the growth of *L. latifolia* plants.

The ethanolic extracts of *M. parviflora* leaves showed higher contents in both total phenolic and flavonoid than found in the aqueous extract, and both extracts possessed strong anti-oxidant properties and were considered good sources of natural products^61^. The aqueous extract of *M. parviflora* showed a total phenol content of 32.95 mg of GAE/g extract^51^. Another study observed that the total phenolic and flavonoids contents were 10.98 mg GAE/g extract and 5.64 mg catechin/g extract, respectively^62^. Caffeic acid, catechin, chlorogenic acid, cinnamic acid, gallic acid, methyl gallate, naringenin, pyrocatechol, rutin and syringic acid were identified in the alcoholic extract of *M. parviflora* leaves^62^.

Furthermore, other reports indicated that the plant is abundant in flavonoids, phenolic compounds, and salicylic acid. It also contains a variety of active ingredients, including anthocyanin, ascorbic acid, asparagine, flavonol glycosides, pectin, phenolic acid, quercetin, salicylic acid and vitamins A, B, and C^63,64^. Organic fertilizer and vermicompost applications have higher phenolic and flavonoid contents in coriander plant^65^. By HPLC, the phytochemical screening detected the constituents kaempferol and apigenin in the leaf extract of *M. parviflora*^66^. Additionally, the bioactive compounds apigenin-7-glucoside, cinnamic acid, luteolin, *ρ*-coumaric acid and naringenin were detected in the *M. parviflora* leaf extracts by HPLC^67^. Because these compounds can scavenge free radicals through a single-electron transfer mechanism, naturally occurring phenols have an anti-oxidant action that reduces oxidative stress in living cells and tissues^68^. catechin, chrysin, apigenin-7-glucoside, cinnamic acid, ferulic acid, gentisic acid, kaempferol, Luteolin, naringenin, *ρ*-coumaric acid, rosmarinic acid and vanillic acid identified as the major polyphenol components in *M. parviflora* leaf extracts, can scavenge DPPH radicals and may be a useful natural source of bioactive chemicals^46,61,69^.

There is little information about using *M. parviflora* leaf extracts as plant-derived biostimulants (PDBs). 10% ME and a blend of 10% ME and 20% Artemisia extracts increased the growth, yield, and protein content of cowpeas beside enhancing soil fertility^70^. PDBs are used to enhance the physiological, biochemical, and molecular processes of several horticulture crops. They boost the post-harvest quality of fruits even in the face of environmental stress by increasing the amount of nutrients that are available in the soil^19,71–73^. Nutrients, amino acids, peptides, peptones, or proteins in the PDBs may all directly contribute to increased nutrient availability^74–76^. PDB treatment may also result in enhanced nitrogen and carbon metabolism and up-regulation of photosynthesis^22,76,77^. Increased sterol levels that control membrane integrity and delayed photo-inhibition have also been linked to the stress tolerance offered by PDBs^75^.

Additionally, a wide range of agronomic techniques are employed to enhance soil qualities and boost plant quality and yield. Applying humic acid (HA) to the soil is one of these agronomic techniques. The organic compounds known as HAs improve the chemical and biological characteristics of the soil and the root environment^78,79^. The fresh and dry herb weight and essential oil (EO) rates of *Origanum syriacum* and *Thymus vulgaris* increased with increasing HA doses^80,81^. These PDBs are combined with other organic fertilizers to enhance fertilizer utilization rate, thereby increasing photosynthetic efficiency^82^. For example, the fresh yield, dry yield, dry leaf yield, and EO content of *Origanum vulgare* all increased as the HA dosage was raised^83,84^. Under salinized or non-salinized irrigation water, foliar application of HA at 1000 mg/L dramatically increased plant growth, flowering capacity, nutritional status, proline accumulation, and chlorophyll index of *Pelargonium peltatum*^85^. The *T. vulgaris* plant showed the highest values for plant height, number of branches, total dry weight, total phenols, and flavonoids when the foliar application of HA was done at a rate of 2 g/L^86^. The fresh weight of basil plants treated with HA increased consistently as compared to the control group. The highest yield was obtained at a concentration of 100 mg/L of HA^87^.

The main compounds of *L. latifolia* EOs, α-pinene, *β*-fenchol, *β*-caryophyllene oxide, β-pinene, camphene, camphor, eucalyptol, Δ-elemene, germacrene D-4-ol, isoborneol, oleic acid methyl ester, and tau.-cadinol as well as were reported in different concentrations according to the treatments used in HA and ME and their combinations. It was characterized by a high percentage of the monoterpene fraction, dominated by the oxygenated ones. 1,8-Cineole (eucalyptol) and camphor were the main constituents of the leaf EOs (on average, 50.40 and 37.3%, respectively)^12^. The monoterpene hydrocarbons and the sesquiterpene fraction ranged from 0.7 to 1.3% and from 0.3 to 0.8%, respectively, in leaf EO from plants grown in Valencia, Spain^12^.

The most abundant components identified in *L. latifolia* from Madrid, Spain, were linalool (35–51%), eucalyptol (26–32%), camphor (10–18%), *α*-pinene (1–2%), *α*-terpineol (1–2%), and α-bisabolene (1–2%)^14^. *γ*-Terpinene (26.8%), camphor (13.8%), and 1,8-cineole (10.2%) were determined as the major compounds from *L. latifolia* Medik^88^. The total extraction yield of the most bioactive EO components of basil showed a positive effect, with eugenol, eucalyptol, and geranyl acetate showing progressive increases in abundance with increasing HA concentrations^87^.

The morphological, biochemical (phenolic compounds and essential oils), and antioxidant characteristics of plants are significantly impacted by fertilizer applications^65^. According to recent research, humic matter’s hydrophobic properties, which are linked to its conformational behavior and possible release of aromatic and phenolic components, may play a role as molecular bioeffectors^89–91^. Increased EO yields of Spike Lavender with lower quality were obtained by soils with increased organic matter and mineral content^92^.

## Conclusion

Applying humic acid to the soil and foliar application of *Malva parviflora* leaf extract had a significant impact on the quality of *Lavandula latifolia* plants as measured by vegetative growth parameters, essential oil chemical compositions, and bioactive ingredient content. The goal of attaining optimal quality through farming methods requires particular attention. We might suggest using humic acid (4 g/L) and *Malva parviflora* leaf extract (4 g/L) in conjunction for the best essential oil percentage and vegetative development. Therefore, despite the existing progress, there is still room to improve understanding in this area. This can be done, for example, by utilizing varying concentrations to examine the effects of exposure times with various environmental variables based on temperature and relative humidity indices.

## Data Availability

All data generated or analyzed during this study are included in this published article.
